# Microsatellite Instability Assay as a Potential Approach to Evaluate Genotoxicity: Lead Exposure in a Nestling Passerine Bird at the Stage of Intensive Erythropoiesis

**DOI:** 10.3390/ani13081325

**Published:** 2023-04-12

**Authors:** Marcin Markowski, Adam Kaliński, Jarosław Wawrzyniak, Michał Glądalski, Joanna Skwarska, Jerzy Bańbura

**Affiliations:** Department of Experimental Zoology and Evolutionary Biology, Faculty of Biology and Environmental Protection, University of Lodz, Banacha 12/16, 90-237 Lodz, Poland

**Keywords:** microsatellite instability, lead intoxication, experimental exposure, passerines, nestlings, biomarkers

## Abstract

**Simple Summary:**

This is the first case of an experimental study with a novel approach to analyze microsatellite instability (MSI) as a detection tool for genetic alterations in a wild population of birds, in this case for great tits (*Parus major*) being exposed to lead compounds. Although this preliminary study did not find any MSI in the analyzed microsatellite markers, it contributes to the examination of this molecular technique as a potentially applicable bioindication tool in ecotoxicological bird studies. We believe that certain issues should be considered in finding an explanation for our result. First, the exposure to lead was too low to induce genetic alterations. Second, the panel of microsatellite markers studied may have been unsusceptible to lead genotoxicity. Third, the time interval (5 days) between lead exposure and blood sampling for genetic analyses were too short to trigger genotoxicity. Further analyzes are needed to verify these findings and to evaluate the scope of application of the MSI analysis in wild bird population studies.

**Abstract:**

Although many avian studies have investigated the toxic effects of lead on important biochemical and physiological processes, organ and system function, and behavior, studies evaluating the specific genotoxic effects of exposure to lead are scarce. Nowadays, rapid technological advances can provide new molecular techniques in this regard. In this study, as a novel approach in bird studies, we used a panel of ten microsatellite loci to investigate the microsatellite instability (MSI) in response to experimental lead intoxication in a common hole-nesting species, the great tit *Parus major*. For this purpose, an experiment based on an intentional single supplementation of a lead (II) acetate trihydrate compound was conducted, with the use of two different doses, applied to randomly chosen great tit nestlings from randomly selected broods, being at the stage of intensive erythropoiesis. Although this preliminary study did not find any MSI in the seven microsatellite markers retained for the final comparison, it contributes to the examination of this molecular technique in field conditions as being potentially applicable in ecotoxicological bird studies. We believe that certain issues should be considered in finding an explanation for our result. First, the single doses of lead used in this study may have been too weak to induce genetic instability. Second, the panel of microsatellite markers studied may have been unsusceptible to lead genotoxicity in general. Third, the relatively short time interval (5 days) between the experimental procedure (lead exposure) and the sampling of post-exposure material (blood) for genetic analyses could have limited the effect of lead genotoxicity. Further analyzes are needed to verify these findings and to evaluate the scope of application of the MSI analysis in wild bird population studies.

## 1. Introduction

In numerous avian studies, lead (Pb) intoxication has been proven to cause a wide range of negative effects, starting from slight disruptions to physiological and biochemical processes, through behavioral alterations, to substantial damage or functioning impairment of meaningful organs and systems. As a consequence, individual conditions evaluated using various environment–sensitive physiological and biochemical indicators, as well as the survival of birds, have been significantly affected in many cases [1,2,3,4,5,6,7]. 

Lead and lead compounds have also been studied for many years in various biological systems as potential mutagenic, clastogenic, and carcinogenic agents [8]. In most clinical and/or laboratory studies of the genotoxic consequences of lead exposure conducted so far, the focus has been on several genetic endpoints as sorts of bioindicator [8,9,10,11,12]. Interestingly, relatively little attention has been paid so far to wild bird populations in this regard. Although several molecular techniques have been successfully employed in avian studies to evaluate molecular alterations induced by various genotoxic contaminants [13,14,15,16,17,18,19,20,21,22,23,24,25,26,27,28,29,30], there is a paucity of work on novel molecular techniques that would allow us to verify the genotoxic properties of lead. Microsatellite instability (MSI) could be potentially effective as such a novel method of detection of genotoxicity of various compounds present in the environment.

Microsatellites, Short Tandem Repeats (STRs), or Simple Sequence Repeats (SSRs) are simple sequences that contain a series of repeats of one to six nucleotide base pair motifs. They are a characteristic component of the nuclear genome of most eukaryotic organisms and are characterized by a high level of polymorphism. At the same time, they are considered as important markers of genomic DNA damages, e.g., in deletions or insertions and/or point mutations [31]. Microsatellite instability analyses in a relatively small number of clinical and animal laboratory studies has confirmed that exposure to selected heavy metals, including lead and its compounds, can induce MSI [31,32,33,34,35,36,37,38]. For example, the effect of cadmium on genomic stability was analyzed for laboratory outbred strain mouse models. Using a panel of six microsatellite markers, Oliveira et al. [36] tested whether a single subcutaneous injection of CdCl_2_ induces MSI in murine testes. It turned out that the lowest dose out of three used in this study triggered the size alteration of two microsatellite loci. In another similar study, Du et al. [37] tested the mutagenic effects of cadmium on 42 SSRs in murine liver, kidney, testis, leukocytes, bone marrow, and small intestine. That study confirmed the presence of MSI at three microsatellite loci in the kidney and in leukocytes of individuals exposed to lower doses of cadmium chloride.

Genomic instability is also important from the viewpoint of human exposure to heavy metals, as it may lead to the acquisition of cancer hallmarks. Zienolddiny et al. [32], using two lines of human lung tumor cells, studied the potential genotoxic effects of nickel sulfate. As a result of the conducted analysis, the authors found that nickel can promote SSRs mutations in the majority of cell line samples. A correlation between heavy metal exposure and MSI has also been evaluated in traditional clinical studies. As one of the aims, Wang et al. [38] conducted such an analysis on 105 recruited patients with pathologically confirmed gastric cancer and 62 health controls. MSI was detected in 18 patients with cancer. The concentration of 13 out of 18 heavy metals differed significantly between healthy and cancer patients. Among the trace elements, gallium was found in significantly higher amounts in the group of patients with confirmed MSI compared to the patients with stable microsatellites, and only stibium had a positive correlation with MSI. Wang et al. [38] concluded that stibium has the potential to induce MSI in humans with gastric cancer. In another study, Hirose et al. [33] analyzed genomic instability using a panel of six microsatellite loci for chromate-exposed workers with lung cancer. The authors found that an increase in the time of exposure to the chromate was significantly associated with a higher frequency of MSI. Their findings suggest that MSI could be relevant in chromium-induced carcinogenesis.

To the best of our knowledge, the effect of any toxic heavy metal including lead on microsatellite instability has not yet been examined for wild bird species. As birds are characterized by a short lifespan of red blood cells in general—and in the case of developing nestlings, erythrocytes are being intensively produced from stem cells—the detection of replication errors, including those in microsatellites, seems likely. Therefore, avian blood has been recently considered to be a good matrix to assess genotoxic alterations reflecting recent exposure effects [28]. Assuming that lead exposure may cause replication errors in erythrocyte stem cells in the bone marrow of birds, newly emerging erythrocyte lines may show altered microsatellite genotypes as indicators of the MSI.

Hence, as a novel approach, we decided to verify the possibility of implementing the MSI method to examine the genotoxic effect of lead intoxication in a common hole-nesting species, the great tit *Parus major*, at the stage of intensive erythropoiesis. For this purpose, we conducted an experiment on nestlings, based on a single supplementation of lead acetate solution, with two dose levels. We applied a set of microsatellite markers to examine and compare the allele size among individuals exposed to the specified doses of lead and those that were assigned to the control group. Our basic goals are (i) to conduct a preliminary assessment of the application of the MSI analysis for a wild passerine species (the great tit) and (ii) to verify if the selected microsatellite loci could be used as markers of exposure to toxic agents (in this particular case, of exposure to lead).

## 2. Materials and Methods

### Study Site, Experiment Layout, Sampling, Isolation, Genotyping, and Statistical Analysis

This experimental study was carried out as part of our long-term research concerning the breeding biology of hole-nesting passerines in central Poland [39]. The study was conducted in accordance with recommendations reported in the ARRIVE guidelines. All performed procedures in the study fulfilled the criteria of Polish legislation and were approved by the Local Bioethical Commission for Experiments on Animals, Medical University in Lodz (No. 70/ŁB07/2015 and 11/ŁB07/2016) and the Regional Directorate for Environmental Protection (WPNII.6401.13.2016.MS, WPNII.6401.122.2016.KW2).

**Figure 1:**
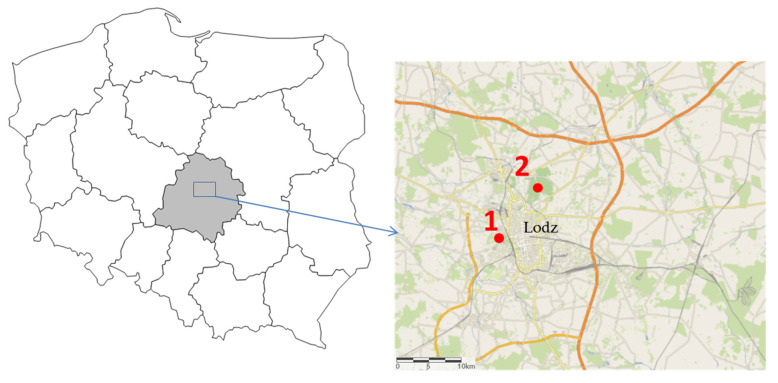
Study area with the spatial location of the study sites (1—urban parkland; 2—mature deciduous forest). The map of Poland was created with STATISTICA software [41] using the Maps add-on (v. 3.1.85). The map with the pointed study sites was made by applying QGIS 3.16.3 Hannover software (https://www.qgis.org/, accessed on 1 September 2022). Both maps were merged and modified in GIMP v. 2.10.30 (GIMP Development Team 2022: http://gimp.org, accessed on 1 September 2022).

During the 2021 breeding season, an experiment was carried out on 20 randomly selected broods of great tit, which included 10 broods from two study sites representing two types of contrasting habitats around Lodz, central Poland (urban park and mature deciduous forest; Figure 1). A more detailed description of the study sites can be found in [40].

The experiment was based on a previously designed and described setup [6], in which the nestlings were intentionally exposed through the gape to a solution of lead (II) acetate trihydrate, in doses which simulated increased levels of lead in the environment but which did not have a lethal effect. From each brood included in this experiment, 6 individuals on the 10th day of their lives were randomly selected and individually banded by using unique numbered aluminum rings. Samples of ca. 10 µL of blood per nestling were collected from the ulnar vain and applied onto FTA MiniCards, which after drying were stored at room temperature ready for the subsequent genetic analyses. Then, four randomly selected nestlings were administered a single dose of lead acetate solution: two of them received a dose of 15 µg Pb/g of body weight and the other two were administered a dose of 30 µg Pb/g of body weight. The remaining two nestlings were treated as controls and received deionized water as a placebo. On the 15th day of the nestlings’ lives, blood was again sampled onto the FTA MiniCards in the same layout. The lead doses used in this study were selected on the basis of our previous research [6,42] and following the relevant literature [43]. It was assumed that the lead doses applied simulated the exposure to lead and corresponded to lead burdens for great tits in areas considered to be highly contaminated (a dose of 30 μg Pb/g body weight) and minorly contaminated (a dose of 15 μg Pb/g body weight) [43].

All individuals included in this experiment were genotyped at ten microsatellite loci (PmaC25, PmaD22, PmaD130, PmaGAn27, PmaGAn30, PmaGAn40, PmaTAGAn71, PmaTAGAn86, PmaTGAn42, and PmaTGAn45), primarily developed for great tits, mainly for the purpose of use in population genetic and evolutionary studies [44].

To extract DNA from the gathered samples, the GeneMATRIX Bio-Trance DNA Purification Kit was used. In the first step, a part of dried blood sample (ca. 2 mm^2^) was cut out from each FTA MiniCard by using a sterile cutter. Then, exact isolation procedure was conducted in accordance with the manufacturer’s instructions. PCR assay was performed in a 20 µL volume containing 10 µL of DreamTaq PCR MasterMix (Thermo Fisher Scientific Inc., Waltham, MA, USA) and 0.5 µL of each primer. A total of 1 µL of the extracted DNA was added to each reaction sample. PCR amplifications followed the steps and conditions presented in the protocol developed by [44]. Forward primers used in PCR were labeled with 6-FAM fluorescent dye. Microsatellite genotyping was performed in a commercial laboratory (Genomed, Warsaw, Poland). Sizes of the alleles were scored against an internal lane size standard (Genescan TM 600 LIZ, v2.0, Applied Biosystems, Foster City, CA, USA) by applying Geneious v11.1.5 software (Biomatters Ltd., Auckland, New Zeland).

To examine any mistyped allele sizes and scoring errors due to large allele dropout or stuttering (i.e., the failure to discriminate similar-sized alleles in genotypes due to PCR artifacts), the Micro-Checker 2.2.3 software was used [45]. While for all individuals, most loci gave consistent and successful genotyping, three of them (PmaGAn30, PmaTAGAn86, and PmaTGAn45) were unreadable, mostly due to amplification problems. Consequently, we retained a panel of seven out of ten microsatellite loci (Table 1) in the further analyses.

Statistical analyses were performed using STATISTICA 13.0 software [41]. The results are reported as means ± standard error (S.E.). Since all data did not meet the assumption of normal distribution, the Mann–Whitney U test was applied to examine the overall differences in allele sizes for each of the seven microsatellite loci of the same nestlings sampled and genotyped twice. These analyses were conducted separately for individuals assigned to one of the three experimental groups. Statistical significance was defined as *p* ≤ 0.05.

## 3. Results and Discussion

Exposure to widely distributed environmental pollutants, such as lead, adversely affects various groups of organisms. Lead has been proven to have toxic properties in most of its chemical forms, which have a wide range of detrimental effects on the physiological, behavioral, and biochemical functions of animals. Lead triggers deleterious effects on the haematopoietic, vascular, nervous, renal, reproductive, and immune systems, mainly through the increase in oxidative stress [46,47,48,49,50,51,52,53].

In fish, exposure to lead induces oxidative stress generated through the excessive production of reactive oxygen species (ROS). As a consequence, the neurotoxicity effect may occur, resulting in synaptic damage and the malfunction of neurotransmitters, leading to behavioral and cognitive dysfunction [53]. Lead was also described as an immune-toxicant factor inhibiting the immune response of fish. According to Dunier [54], Pb decreases haematopoietic activity in the spleen, as well as limiting phagocytic activity and antibody production.

In the case of amphibians, several signs of lead intoxication have been reported. Although mostly concerning mortality after exposure [55], some studies have focused on alterations in development. Perez-Coll et al. [56] showed that in Pb-exposed embryos of the toad *Bufo arenarum,* stunted tails, underdeveloped gills, microcephaly, and increased mortality occurred. In another study, conducted on the tadpole *Xenopus laevis* [57], lead was also identified as a detrimental factor responsible for spinal deformities.

Compared to other vertebrates, there are fewer toxicological studies on reptiles, particularly those that analyze the toxic effect of lead. Nevertheless, there are some surveys that provide knowledge in this respect. Salice et al. [58] proved that the Western fence lizard *Sceloporus occidentalis*, when exposed to increased doses of lead, experienced reduced survival and decreased growth and food consumption. Hematological parameters such as haematocrit and haemoglobin concentration decreased significantly with increased exposure to lead, which points to the classic signs of anemia that Pb induces.

The toxic effects of lead poisoning have been widely studied in mammals. In this regard, studies on humans provide valuable data on lead toxic properties for this group of vertebrates. The main targets for Pb are mammals’ erythrocytes and their precursors, as well as the kidneys and the central and peripheral nervous system [59]. As a consequence, in many cases chronic exposure to lead has been reported to induce anemia and an increase in blood pressure. High levels of lead were recorded as a significant factor of severe damage to the brain and kidneys, regardless of age. Lead toxicity manifested also in the alteration of reproduction. Long-time exposure to high doses of lead caused miscarriages in pregnant woman, the low birth weights of infants, and reduced fertility in males. Intense exposure to lead also affects the central and peripheral nervous system, giving the symptoms of short-term memory loss, reduced concentration, depression, headaches, numbness, tingling in the arms and legs, and problems with coordination [60]. The neurotoxic effects of lead were also recorded in rats and monkeys exposed to lead during the prenatal, post-natal, and adult life stages. Studies on small mammals such as the bank vole, wood mouse, and shrews found that lead intoxication induced kidney damage [61].

Among all vertebrates, birds are pointed out as the most studied and very likely most affected taxon in regard to lead poisoning as an effect of exposure to contaminated food, mainly by lead shot pellets and/or lead fishing sinkers [6,7]. The character and intensity of clinical signs observed in lead-intoxicated birds notably depend on the degree of exposure, with the time and quantity of absorbed lead being crucial [3]. Accordingly, typical symptoms of chronic exposure to lead include anemia, weakness, ataxia, weight loss, visible difficulties in flying and walking, vomiting and green diarrhea, and convulsions [6,7,62]. According to Pain et al. [7], acute poisoning with high doses of lead can even induce lethal effects for birds without manifesting any of the mentioned signs of its toxicity.

Toxicity of lead has been also investigated with regard to its mutagenic, clastogenic, and carcinogenic properties. However, the gathered data are still equivocal and many studies present contradictory conclusions. Moreover, they mostly concern human, animal, or cell-line studies conducted under clinical and/or laboratory conditions [8,63,64]. In this regard, there is a noticeable paucity of studies that concern investigation of the genotoxic effects of lead on wild living birds. We are aware of only a few that have analyzed the adverse effect of the heavy metal toxic spill from the Aznalcollar mine (southwestern Spain) on populations of two bird species that occupy a high level in the food chain, namely, the white stork *Ciconia ciconia* and black kite *Milvus migrans* [16,17,18,19]. In these studies, the authors applied the comet assay and showed that individuals of both species from the contaminated sites were characterized by an increased level of DNA damage measured in their lymphocytes in comparison to birds tested at the reference sites, although some inter- and intra-species differences were noted. According to Baos et al. [19], lead was not significantly related to DNA damage in either nestling white storks or black kites during the conducted study. Presumably, this was caused by the low number of white stork and black kite nestlings exposed to doses of lead responsible for sublethal effects, 0.4% and 2.3%, respectively. In general, the paucity of bird studies that directly concern the detection and examination of genetic alterations induced by a single selected trace element such as lead offers an opportunity to fill this gap and provide new knowledge in this respect.

**Figure 2:**
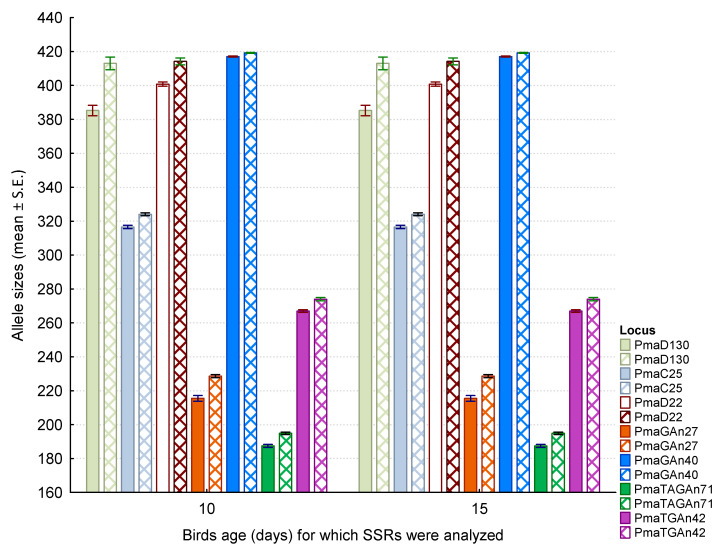
Allele sizes (mean ± S.E.) recorded for SSRs genotyped twice in 5-day interval for nestlings, which were supplemented with a dose of 15 µg Pb/g of body weight.

**Figure 3:**
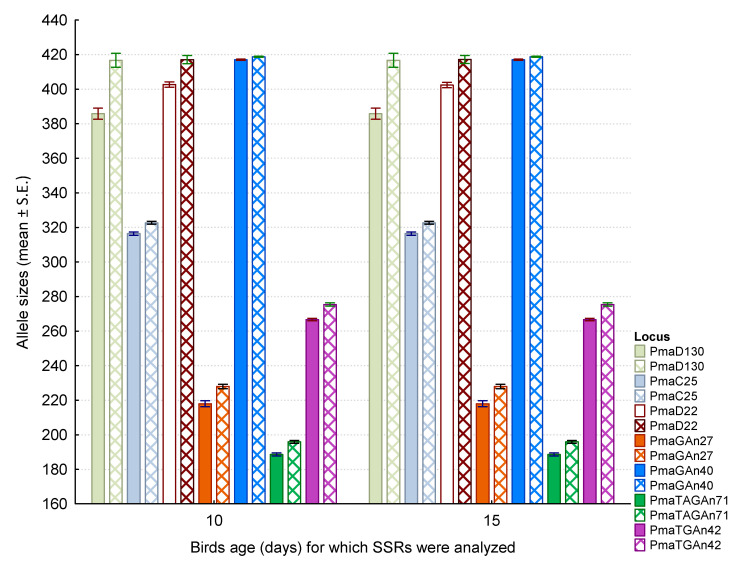
Allele sizes (mean ± S.E.) recorded for SSRs genotyped twice in 5-day interval for nestlings, which were supplemented with a dose of 30 µg Pb/g of body weight.

**Figure 4:**
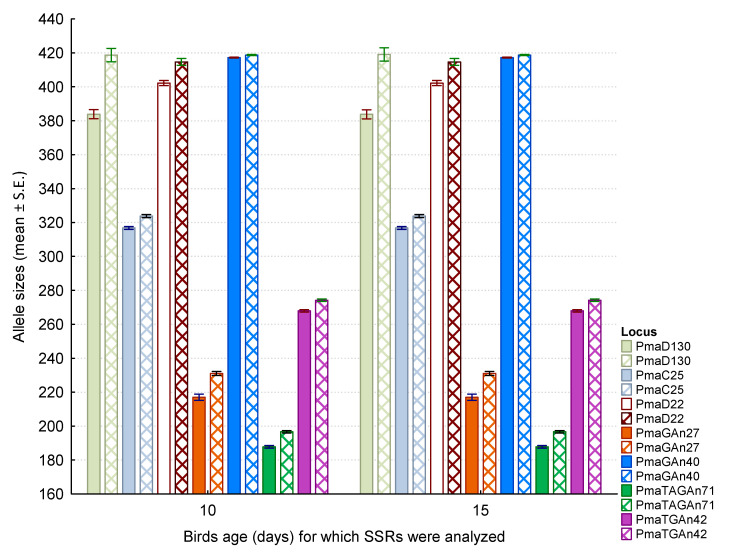
Allele sizes (mean ± S.E.) recorded for SSRs genotyped twice in 5-day interval for nestlings, which were assigned to the control group.

In this study, we did not find any effect of experimental lead intoxication on genomic instability in nestling great tits by using peripheral blood erythrocytes. We found no change in allele sizes (microsatellite instability, MSI) in any of the seven tested loci (PmaC25, PmaD22, PmaD130, PmaGAn27, PmaGAn40, PmaTAGAn71, and PmaTGAn42) in the great tit nestlings that received a dose of 15 µg Pb/g of body weight (Figure 2; Supplementary Table S2; Table 2), nor in nestlings with a dose of 30 µg Pb/g of body weight (Figure 3; Supplementary Table S3; Table 2). Analogously, no alterations in allele sizes were observed for individuals from the control group (Figure 4; Supplementary Table S1; Table 2).

To our best knowledge, this is the first case of an experimental study with a novel approach to analyze MSI as a detection tool for genetic alterations in a wild population of birds. The lack of bird studies on the application of the microsatellite instability assay to test the genotoxicity of heavy metals makes direct comparison of our results impossible. However, we think that referring to some other study systems can be helpful in understanding our negative results [31,35]. Although the selected microsatellite markers did not show any signs of significant response to lead exposure (i.e., alterations in allele sizes), we still believe that such an effect is possible. At the same time, we think that the concept and approach of this study generally may direct attention to the microsatellite instability assay as a molecular technique that is potentially applicable in ecotoxicological bird studies.

We believe that the most likely reason for our negative experimental result was that the single doses of lead acetate administered to nestlings were too low to initiate any of the indirect mechanisms that could exert any modification in the sizes of the SSRs we analyzed. Even though the biochemical and molecular mechanisms underlying the genotoxicity of lead are not yet fully elucidated, it has been reported that lead induces a genotoxic effect throughout indirect mechanisms, such as the inhibition of DNA repair, the excess production of free radicals contributing to DNA damage, and the regulation of suppressor and promotor genes in carcinogenesis [8,12]. Such conclusions were assumed in one of the experimental studies conducted by [35], in which MSI was evaluated using three microsatellite loci located within the genome of rats (*Rattus norvegicus*) exposed to various heavy metal compounds. The authors detected MSI in all of the loci tested for the rats exposed both to a single and to multiple doses of lead acetate and arsenic trioxide used in the study. El-Ghor et al. [35] suggested that oxidative stress or defects in mismatch repair genes could play a pivotal role in the genotoxicity of the metal compounds used, including lead acetate. The study by El-Ghor et al. [35] is the only study published to date to analyze MSI in a vertebrate species under exposure to lead acetate. The explanation of our result also seems to be reasonable because of another study, [31], which is conceptually and methodologically similar to [35], except for a cadmium compound being used instead of lead. El-Ghor et al. [31] found that in the case of single exposure to cadmium chloride, no microsatellite instability was observed in the three tested loci. In contrast, for rats that received multiple oral doses of cadmium, MSIs were recorded in three of the studied loci. To explain these results, the authors also invoke indirect mechanisms that may induce genotoxicity, in particular a mechanism based on cadmium chloride being able to inhibit DNA-mismatch repair.

We are aware that, apart from the fact that the single doses of lead used in this study may have been too weak to induce genetic instability, some other issues should also be considered in finding an explanation for our result. First, the panel of microsatellite markers selected to determine MSI could be unsusceptible to lead genotoxicity in general. Second, the relatively short time interval (5 days) between the experimental lead exposure and the post-exposure blood sampling for genetic analyses could have largely limited the effect of lead genotoxicity. Consequently, our results indicate the need for further, more complex research to investigate whether the single doses of lead acetate as used in this study were too low to induce genetic instability and/or whether a selected panel of microsatellite markers characterize susceptibility to lead genotoxicity in general. In further research, blood lead levels should also be investigated to obtain additional significant data about lead’s toxic effects and to examine how blood lead levels change with exposure time.

## Figures and Tables

**Table 1:** Summary of seven microsatellite loci characteristics for the great tits, including number of individuals (N), allele size ranges, motif of cloned alleles, and primer sequences.

Locus	N	Size Range (bp)	Motif of Cloned Allele	Primer Sequences
PmaD130	119	376–452	(TAGA)_17_	F°:TGAGTGGAAAGATGCTGGC R°:CCCTATAAAAACCGAGGCTG
PmaC25	120	303–330	(CAT)_11_	F°:CGTCCTGCTGTTTGTATTTCTG R°:CCATGAACCATTTTTAGGGTG
PmaD22	120	376–448	(CTAT)_15_(CCAT)_12_	F°:GATCAGAGCTTGCCTCAACAC R°:TCTGGGCTGAAATACCTACCC
PmaGAn27	120	198–253	(CAT)_16_	F°:TATAAACCACAGCCACACGC R°:CACAACCACAGAGGCATGAG
PmaGAn40	120	406–422	(GA)_10_	F°:CGTTCCTCCTTTGCTTTCTG R°:AATGGCACAACACCTTCTCC
PmaTAGAn71	120	173–209	(TAGG)_6_(TAGA)_11_	F°:TCAGCCTCCAAGGAAAACAG R°:GCATAAGCAACACCATGCAG
PmaTGAn42	120	260–292	(TCCA)_15_	F°:ACTTCCACATGCCAGTTTCC R°:TGTTAAGGCAGAGAGGTGGG

F: forward primer; R: reverse primer.

**Table 2:** Results of the Mann–Whitney U test for the comparison of the mean allele sizes of SSRs genotyped twice for the studied individuals, with respect to the dose at which nestlings were supplemented with lead.

	Dose of Lead (μg/g of Body Mass)		
Locus (Two Alleles)	Control	15	30
PmaD130	Z = 0.013; *p* = 0.99	Z = 0.007; *p* = 0.99	Z = −0.007; *p* = 0.99
PmaD130	Z = -0.092; *p* = 0.93	Z = 0.005; *p* = 1.00	Z = −0.005; *p* = 1.00
PmaC25	Z = 0.005; *p* = 1.00	Z = 0.005; *p* = 1.00	Z = −0.005; *p* = 1.00
PmaC25	Z = 0.005; *p* = 1.00	Z = 0.005; *p* = 1.00	Z = −0.005; *p* = 1.00
PmaD22	Z = 0.005; *p* = 1.00	Z = 0.005; *p* = 1.00	Z = 0.226; *p* = 0.82
PmaD22	Z = 0.005; *p* = 1.00	Z = 0.005; *p* = 1.00	Z = 0.000; *p* = 1.00
PmaGAn27	Z = 0.005; *p* = 1.00	Z = 0.000; *p* = 1.00	Z = 0.005; *p* = 1.00
PmaGAn27	Z = 0.005; *p* = 1.00	Z = −0.005; *p* = 1.00	Z = 0.005; *p* = 1.00
PmaGAn40	Z = 0.006; *p* = 1.00	Z = −0.006; *p* = 1.00	Z = 0.006; *p* = 1.00
PmaGAn40	Z = 0.005; *p* = 1.00	Z = −0.005; *p* = 1.00	Z = 0.005; *p* = 1.00
PmaTAGAn71	Z = 0.005; *p* = 1.00	Z = −0.005; *p* = 1.00	Z = 0.005; *p* = 1.00
PmaTAGAn71	Z = 0.005; *p* = 1.00	Z = −0.005; *p* = 1.00	Z = 0.005; *p* = 1.00
PmaTGAn42	Z = 0.005; *p* = 1.00	Z = −0.005; *p* = 1.00	Z = 0.005; *p* = 1.00
PmaTGAn42	Z = 0.005; *p* = 1.00	Z = −0.005; *p* = 1.00	Z = 0.005; *p* = 1.00

## Data Availability

The data presented in this study are available in the Supplementary Materials.

## Supplementary Materials

The following supporting information can be downloaded at https://www.mdpi.com/article/10.3390/ani13081325/s1, Table S1: Allele sizes recorded for SSRs genotyped twice in 5-day interval for nestlings assigned to the control group; Table S2: Allele sizes recorded for SSRs genotyped twice in 5-day interval for nestlings, which were supplemented with a dose of 15 µg Pb/g of body weight; Table S3: Allele sizes recorded for SSRs genotyped twice in 5-day interval for nestlings, which were supplemented with a dose of 30 µg Pb/g of body weight.
